# Recurrent, low-frequency coding variants contributing to colorectal cancer in the Swedish population

**DOI:** 10.1371/journal.pone.0193547

**Published:** 2018-03-16

**Authors:** Xiang Jiao, Wen Liu, Hovsep Mahdessian, Patrick Bryant, Jenny Ringdahl, Maria Timofeeva, Susan M. Farrington, Malcolm Dunlop, Annika Lindblom

**Affiliations:** 1 Department of Molecular Medicine and Surgery, Karolinska Institutet, Stockholm, Sweden; 2 Colon Cancer Genetics Group, Institute of Genetics and Molecular Medicine, University of Edinburgh, Edinburgh, United Kingdom; 3 MRC Human Genetics Unit, Western General Hospital Edinburgh, Edinburgh, United Kingdom; Kunming Institute of Zoology, Chinese Academy of Sciences, CHINA

## Abstract

Genome-wide association studies (GWAS) have identified dozens of common genetic variants associated with risk of colorectal cancer (CRC). However, the majority of CRC heritability remains unclear. In order to discover low-frequency, high-risk CRC susceptibility variants in Swedish population, we genotyped 1 515 CRC patients enriched for familial cases, and 12 108 controls. Case/control association analysis suggested eight novel variants associated with CRC risk (OR 2.0–17.6, p-value < 2.0E-07), comprised of seven coding variants in genes *RAB11FIP5*, *POTEA*, *COL27A1*, *MUC5B*, *PSMA8*, *MYH7B*, and *PABPC1L* as well as one variant downstream of *NEU1* gene. We also confirmed 27 out of 30 risk variants previously reported from GWAS in CRC with a mixed European population background. This study identified rare, coding sequence variants associated with CRC risk through analysis in a relatively homogeneous population. The segregation data suggest a complex mode of inheritance in seemingly dominant pedigrees.

## Introduction

Colorectal cancer (CRC) remains a major source of cancer morbidity and mortality, as it accounts for the fourth most common cause of cancer deaths worldwide [1]. In Sweden 20% of new cases have at least one close relative with CRC and their family members are at increased risk of the disease [2]. Since CRC is considered to develop over precursor lesions detectable and removable in risk-individuals under surveillance, it is important to identify subjects at increased risk [3]. Less than 5% of all CRC represent high-penetrant CRC syndromes, most commonly familial adenomatous polyposis (FAP) or Lynch syndrome [2, 4].

High-risk genes have typically been identified via linkage analysis in large pedigrees or sequencing of candidate genes [5–7], and more recently also by next-generation sequencing (NGS) [4, 8–10]. Genome-wide association studies (GWAS) using chip-based techniques have been applied to study common genetic variants in order to find out the proportion of their contribution to the CRC risk. To date, less than 100 CRC susceptibility variants have been identified across less than 50 chromosomal regions [11–14], however, in total these SNPs and their surrounding regions represent no more than 2% of the heritability of CRC [15], and typically, each with a small increased risk (OR < 2).

Thus, a substantial risk is still to be explained since the genetic contribution to CRC is estimated to 35% [16]. The existence of rare high-risk alleles has been hypothesized [17]. In order to test the hypothesis that CRC risk was associated with variations within gene coding sequence, Illumina exome array was used in association studies. The studies used over 12 000 cases and more than twice as many controls from six different European populations, mostly from UK [18]. Some additional novel common CRC low-risk loci were suggested [18]. There is a substantial variation in the frequency of various single nucleotide polymorphisms (SNPs) between populations and even within populations [19, 20]. The vast majority of this association study included the heterogeneous population in England mixed with the more homogeneous population from Scotland, plus four other European populations [18]. In this work, a different study was designed in order to maximize the likelihood of finding rare high- or moderate risk genes. The new study used a more homogeneous population from Sweden and focused mostly on familial cases.

## Materials and methods

### Study subjects

Two hundred and sixty-two familial CRC cases were first recruited from families undergoing genetic counselling at the department of Clinical Genetics, Karolinska University Hospital, during the years 1990–2010. Another 1 253 cases were from a cohort of more than 3 300 consecutive patients operated on for CRC in 14 hospitals in and around Stockholm and Uppsala between 2004 and 2009 and were included in the Swedish Colorectal Cancer Low-risk Study [2]. FAP and Lynch syndrome were excluded using medical records and our current clinical protocol [2, 21], respectively. Cancer in first- and second-degree relatives and cousins was recorded, and pedigrees for the families of the index-person (the patient) were constructed. All diagnoses in family members, which suggested possible CRC, were verified using medical records or death certificates [2]. Altogether, 936 familial CRC cases, who had at least one first-degree relative with CRC, as well as 579 sporadic CRC cases were included in this study. All patients gave written informed consents in accordance with Swedish legislation and the study was approved by the Regional Ethics Committee in Stockholm, Dnr: 02–489 and 16/24-31/1.

As controls we used 18 560 twins from the Swedish Twin Registry [22] that were genotyped using the same platform during 2014–2015. The majority of twins were from the Child and Adolescent Twin Study in Sweden (CATSS), which is an ongoing longitudinal twin study targeting all twins born in Sweden since 1993. Other studies from the Swedish Twin Registry also had samples genotyped using the same platform, therefore are included in this analysis as well. In short, phenotypic data on cancer had previously been obtained through linking the twins to the Swedish Cancer Registry using the unique person identification number available for all Swedish citizens. Only one twin from each twin pair where none was affected was considered eligible for serving as control in the association analysis.

### Genotyping quality control (QC)

Genotyping of 1 515 CRC samples was performed on Infinium Human Exome BeadChip 12v1.0 (containing 247 870 genetic markers, Illumina Inc., San Diego, CA) and called using the corresponding Illumina GenCall algorithm. 16 748 markers and 31 individuals were excluded due to call rate less than 90%.

18 560 individuals from the Swedish Twin Registry (all available dizygotic twins and one twin in each pair of monozygotic twins) were genotyped using the Illumina Infinium PsychArray-24 BeadChip (containing 588 628 genetic markers, Illumina Inc., San Diego, CA) at the SNP&SEQ Technology Platform in Uppsala, Sweden. Variant calling was performed using Illumina GenCall algorithm for common variants and zCall [23] for rare variants. Genotyping results for 18 193 subjects on 569 211 markers passed the initial pre-processing quality assurance. In further QC, markers were excluded if call rate was < 0.98 (3 827 markers), cross-batch discordance was > 10% (102 markers), with more than one discordant genotype within monozygotic twin pairs (323 markers), deviated significantly from Hardy-Weinberg equilibrium (p-value < 1E-06, 2 399 markers), with allele frequency differed by > 10% (absolute difference) from that of 1000 Genomes European samples and mean GenCall scores < 0.5 (6 markers), significantly associated with more than one genotyping batch (p-value < 5E-08, 35 markers), or on Y-chromosome or mitochondria due to poor calling (1 332 markers). Samples were excluded in case of genotyping success rate < 98% (4 samples), abnormal heterozygosity (autosomal inbreeding coefficient F outside ± 0.2, 7 samples), possible sample contamination (sample relatedness > 6 standard deviations from mean in 1 000 random samples, 14 samples), gender discrepancy between reported and X-chromosome heterozygosity-predicted (22 samples), or evidence of non-European ancestry (> 6 standard deviations from the mean values of the first two principal components in 1000 Genomes European populations, 248 samples). 17 898 (98%) individuals and 561 187 (98%) markers passed QC.

Genotyping data of the two platforms were merged on 223 917 genetic markers that are present on both platforms. 41 markers with call rate < 98% in the merged dataset and 2 234 markers with significant deviation from Hardy-Weinberg equilibrium (p-value < 0.001) were further removed. Population stratification was addressed on the merged dataset using PLINK [24] and 363 individuals with evidence of being population outliers based on multidimensional scaling (MDS) analysis were excluded (S1 Fig). Only one twin from each twin pair where none was affected was considered eligible for serving as control in the association analysis. It was further noticed that 48 CRC samples harbored a higher than expected number of rare variants, which were subsequently excluded. Finally, a total of 13 496 individuals, including 1 388 cases and 12 108 controls remained qualified for the association analysis.

### Statistical analysis

The association between allelic dosage for all variants that passed the stringent quality control procedures and CRC status was calculated using standard case/control association analysis in PLINK (command line: plink—assoc) [24].

### Sequencing validation

All candidate risk variants suggested by the statistical analysis were subjected to Sanger sequencing verification in the respective CRC cases. False risk alleles caused by incorrect genotyping were subsequently removed. Available family members of confirmed risk variant carriers were also tested for the same variant by Sanger sequencing to investigate variant segregation in the family.

## Results

**Fig 1:**
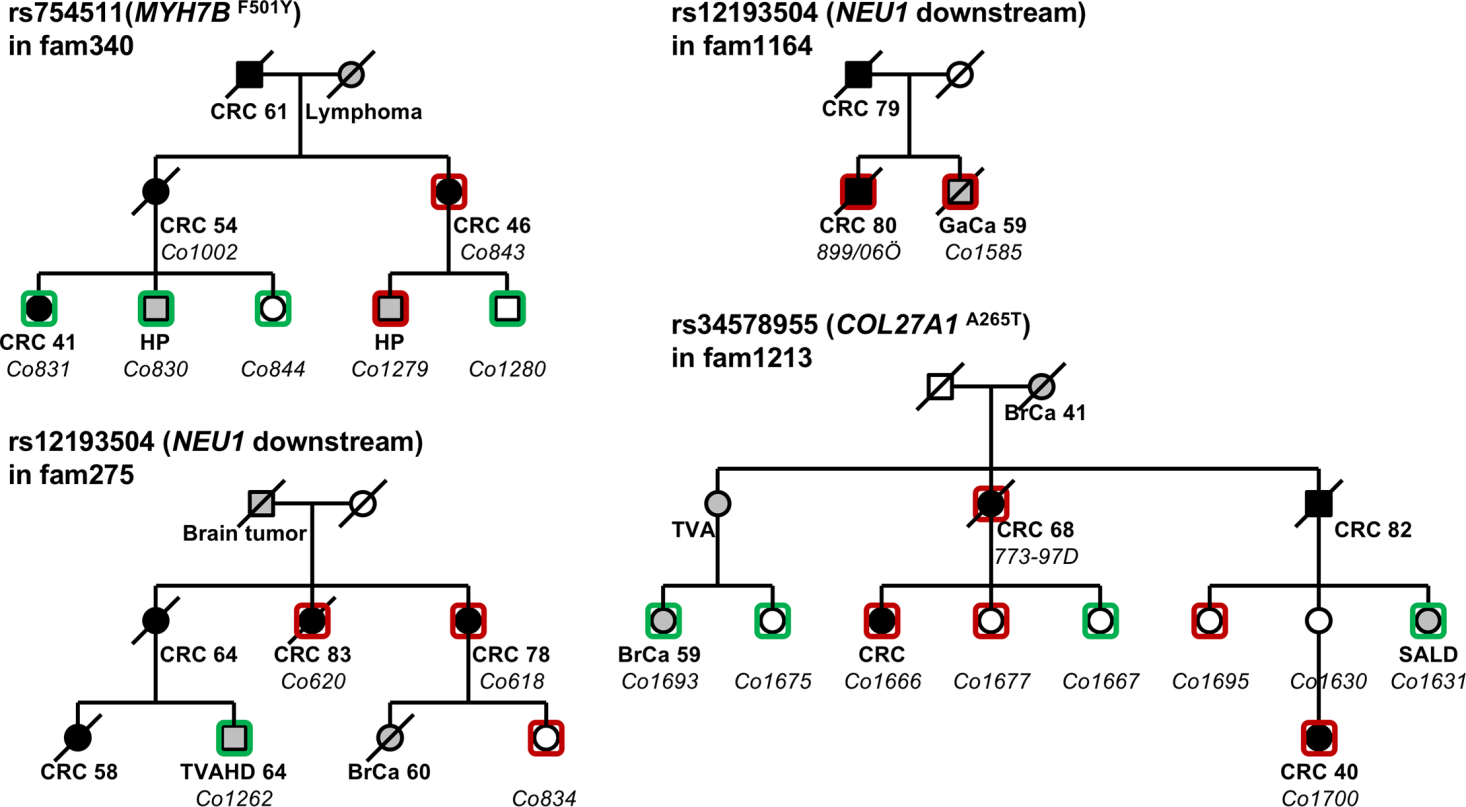
Pedigrees of the families tested for variant segregation. Mutation carriers and non-carriers are indicated with red and green squares, respectively. Diagnosis and age (when available) is indicated under each individual in bold text, and sample IDs in Italic. CRC, colorectal cancer; HP, hyperplastic polyp; GaCa, gastric cancer; TVAHD, tubulovillous adenoma with high degree dysplasia; BrCa, breast cancer; TVA, tubulovillous adenoma; SALD, serrated adenomas with low degree dysplasia.

Post QC exome-wide association analysis was carried out on 1 388 cases and 12 108 controls using 221 642 genetic markers. 79 496 of the markers were monomorphic and 109 616 markers were rare variants (minor allele frequency (MAF) < 1%). The experiment was set up to search for rare alleles with an increased risk of CRC. Thirty-nine markers were observed with the minor allele associated with a statistically significantly elevated risk exceeding Bonferroni-corrected exome-wide threshold (p-value < 2.24E-07). Seven markers were removed due to cross-platform base-calling errors, and Sanger sequencing was performed on all suggested mutation carriers of the remaining 32 markers and confirmed the genotyping results of eight variants (Table 1). All the eight variants were rare (allele frequency < 1% in controls) and associated with moderate risks (OR > 2). Seven of the eight variants were missense variants in genes *RAB11FIP5*, *POTEA*, *COL27A1*, *MUC5B*, *PSMA8*, *MYH7B*, and *PABPC1L*, whereas the other was a downstream variant close to the gene *NEU1* (Table 2). Six of the exonic variants were predicted to be pathogenic by at least one functional inference tool (Table 2). Three of the SNPs could be tested in available family members in four families and segregated more or less with CRC or adenomas in the families (Fig 1).

**Table 1:** Candidate risk variants identified in GWAS confirmed by Sanger sequencing.

SNP	Chr	Position	Ref allele	Risk allele	Allele count Risk / Ref		Risk allele frequency		OR	P-value	MAF				
					Case	Control	Case	Control			SweGen	ExAC All	ExAC NFE	1000G All	1000G EUR
**rs148220987**	2	73315364	C	A	13 / 2763	18 / 24188	0.47%	0.07%	6.3	6.5E-09	0.4%	0.18%	0.11%	0.1%	0.4%
**rs12193504**	6	31820805	G	A	42 / 2714	180 / 23796	1.52%	0.75%	2.0	3.2E-08	1.6%	-	-	5.3%	3.8%
**rs202238834**	8	43152442	A	G	3 / 2687	0 / 24216	0.11%	0%	-	2.0E-07	-	0.01%	0.01%	-	-
**rs34578955**	9	116930628	G	A	4 / 2768	0 / 24216	0.14%	0%	-	3.4E-09	0.05%	0.53%	0.03%	1.5%	0.1%
**rs200554635**	11	1267115	C	A	8 / 2766	20 / 24196	0.29%	0.08%	3.5	3.6E-08	0.1%	0.23%	0.35%	0.2%	0.6%
**rs137990346**	18	23731909	A	G	4 / 2772	0 / 24216	0.14%	0%	-	3.5E-09	0.1%	0.03%	0.05%	0.04%	0.1%
**rs754511**	20	33575677	T	A	29 / 2747	70 / 24132	1.04%	0.29%	3.6	4.5E-10	1.1%	0.63%	1.02%	0.3%	1.3%
**rs201302413**	20	43547582	A	C	10 / 2752	5 / 24211	0.36%	0.02%	17.6	5.5E-13	0.3%	0.27%	0.31%	0.04%	0.1%

MAF was extracted from the SweGen (https://swegen-exac.nbis.se/), ExAC (http://exac.broadinstitute.org/) and 1000Genomes (http://www.internationalgenome.org/) databases (date of access: 2017-11-07). NFE, non-Finnish European; EUR, European.

**Table 2:** Functional prediction of validated risk variants.

SNP	Gene	AA change	PolyPhen [25]	SIFT [26]	LRT [27]	MutationTaster [28]	Mutation Assessor [29]	FATHMM [30]
**rs148220987**	*RAB11FIP5*	R461L	Probably damaging	Tolerated	Deleterious	Disease causing	Medium	Tolerated
**rs12193504**	*NEU1* (Downstream)	-	-	-	-	-	-	-
**rs202238834**	*POTEA*	Q143R	Benign	Tolerated	Neutral	-	Neutral	Tolerated
**rs34578955**	*COL27A1*	A265T	Benign	Tolerated	-	Polymorphism	Medium	Damaging
**rs200554635**	*MUC5B*	T3002K	Probably damaging	NA	-	Polymorphism	Low	Tolerated
**rs137990346**	*PSMA8*	V112A	Possibly damaging	Damaging	Deleterious	Disease causing	Medium	Tolerated
**rs754511**	*MYH7B*	F501Y	Probably damaging	Tolerated	Neutral	Disease causing	Medium	Damaging
**rs201302413**	*PABPC1L*	E180A	Possibly damaging	Damaging	Deleterious	Disease causing	Low	Damaging

We compared our results with the findings in a previous study [18]. Among the 30 previously reported risk SNPs comprising of 15 statistically significant markers and 15 markers with less evidence, 27 were confirmed with an increased risk (OR > 1), including all 15 significant risk variants and 12 out of 15 variants that did not reach the significance threshold (Table 3).

**Table 3:** Comparison of previously reported risk variants [18] to the current study.

SNP	Position	Gene	Ref allele	Risk allele	Previous report [18]				This study			
					Freq in case	Freq in ctrl	OR	P-value	Freq in case	Freq in ctrl	OR	P-value
**rs6687758**	1:222164948		A	G	0.22	0.2	1.1	3.2E-11	0.23	0.20	1.17	8.8E-04
**rs16892766**	8:117630683		A	C	0.1	0.08	1.3	3.6E-17	0.11	0.10	1.16	1.7E-02
**rs16888728**	8:117783975	*UTP23*	G	A	0.11	0.1	1.2	1.4E-07	0.11	0.11	1.03	6.9E-01
**rs10505477**	8:128407443		G	A	0.55	0.51	1.2	2.1E-21	0.53	0.51	1.11	1.0E-02
**rs6983267**	8:128413305		A	C	0.56	0.52	1.2	1.1E-27	0.54	0.52	1.10	2.0E-02
**rs7014346**	8:128424792		G	A	0.41	0.37	1.2	4.2E-24	0.38	0.36	1.12	5.8E-03
**rs6580742**	12:50727811	*FAM186A*	G	A	0.2	0.19	1.1	1.2E-07	0.18	0.17	1.07	1.7E-01
**rs12303082**	12:50754563	*FAM186A*	C	A	0.37	0.35	1.1	7.4E-08	0.34	0.32	1.09	3.8E-02
**rs1129406**	12:51203371	*ATF1*	G	A	0.43	0.4	1.1	8.3E-09	0.40	0.38	1.08	5.9E-02
**rs3184504**	12:111884608	*SH2B3*	A	G	0.53	0.51	1.1	3.9E-07	0.56	0.54	1.11	1.1E-02
**rs4779584**	15:32994756		G	A	0.21	0.19	1.2	2.3E-18	0.22	0.19	1.22	4.4E-05
**rs4939827**	18:46453463	*SMAD7*	G	A	0.57	0.52	1.2	1.3E-33	0.54	0.50	1.18	6.3E-05
**rs10411210**	19:33532300	*RHPN2*	A	G	0.92	0.91	1.2	2.4E-08	0.92	0.91	1.12	1.4E-01
**rs961253**	20:6404281		C	A	0.39	0.36	1.1	6.8E-12	0.39	0.36	1.13	2.6E-03
**rs4925386**	20:60921044	*LAMA5*	A	G	0.71	0.68	1.1	8.7E-10	0.73	0.69	1.20	4.0E-05
**rs78446341**	2:160690656	*LY75*	G	A	0.03	0.02	1.3	3.3E-05	0.01	0.01	1.18	3.3E-01
**rs6599132**	3:41039907		A	G	0.57	0.55	1.1	2.5E-05	0.54	0.53	1.04	2.8E-01
**rs2548145**	5:40134777		A	G	0.54	0.52	1.1	6.9E-05	0.52	0.52	0.97	4.1E-01
**rs2282978**	7:92264410	*CDK6*	A	G	0.34	0.32	1.1	1.1E-06	0.33	0.33	1.03	4.8E-01
**rs6580741**	12:50727706	*FAM186A*	C	G	0.37	0.35	1.1	3.9E-05	0.34	0.32	1.10	3.3E-02
**rs7296291**	12:50744119	*FAM186A*	A	G	0.37	0.35	1.1	5.8E-05	0.34	0.32	1.09	4.0E-02
**rs11169552**	12:51155663		A	G	0.75	0.73	1.1	2.6E-05	0.71	0.69	1.07	1.0E-01
**rs861204**	12:51237816	*TMPRSS12*	A	G	0.67	0.66	1.1	4.2E-05	0.68	0.68	1.00	9.6E-01
**rs10774625**	12:111910219	*ATXN2*	A	G	0.52	0.49	1.1	1.1E-05	0.55	0.52	1.13	2.1E-03
**rs653178**	12:112007756	*ATXN2*	G	A	0.53	0.51	1.1	1.7E-06	0.56	0.54	1.12	4.7E-03
**rs7315438**	12:115891403		G	A	0.59	0.57	1.1	3.0E-05	0.63	0.61	1.09	5.0E-02
**rs11869286**	17:37813856	*STARD3*	G	C	0.34	0.32	1.1	7.3E-05	0.33	0.33	1.03	4.9E-01
**rs2307019**	19:49244220	*IZUMO1*	A	G	0.59	0.58	1.1	6.1E-05	0.58	0.57	1.04	3.9E-01
**rs2236200**	20:60986019	*C20orf151*	C	A	0.76	0.74	1.1	3.6E-05	0.80	0.76	1.23	2.6E-05
**rs1209950**	21:40173528		G	A	0.43	0.41	1.1	7.3E-06	0.40	0.40	0.98	6.8E-01

To further study the possibility that the eight SNPs are not pathogenic *per se*, but rather associated with other pathogenic SNPs in adjacent regions, we selected eight additional markers among all SNPs located within 1 Mb distance from the eight original risk alleles based on their risk and significance (p-value < 3E-05) for further investigation. Sanger sequencing confirmed the genotyping results in three of the eight markers, located in genes *ALMS1*, *COL27A1* and *HNF4A*, respectively (Table 4).

**Table 4:** Additional candidate risk variants located within 1 Mb from the eight original risk markers.

SNP	Chromosomal band	Position	Ref allele	Risk allele	Gene	AA change	Freq in case	Freq in ctrl	OR	P-value	MAF SweGen	MAF 1000G	MAF ExAC
**rs202114540**	2p13.1	2:73677049	A	G	*ALMS1*	Q1131R	7.2E-4	0	-	3E-05	-	NA	5.0E-05
**rs140849642**	9q32	9:117027755	G	A	*COL27A1*	splice region	7.2E-4	0	-	3E-05	-	NA	1.8E-04
**rs201749293**	20q13.12	20:43043289	G	A	*HNF4A*	P212L	7.2E-4	0	-	3E-05	5E-04	NA	3.1E-04

MAF was extracted from the SweGen (https://swegen-exac.nbis.se/), ExAC (http://exac.broadinstitute.org/) and 1000Genomes (http://www.internationalgenome.org/) databases (date of access: 2017-07-25).

## Discussion

Our previous study, testing a heterogeneous population of unselected CRC patients and controls with the Illumina exome chip, confirmed some of the more common risk alleles and was able to suggest 30 new ones, with frequencies between 10–50% and ORs typical for the low-risk allele pattern of 1.1–1.3 [18]. The present study interrogating a more homogeneous population with a selection of mostly familial CRC patients demonstrated eight novel rare moderate-risk loci confirming the hypothesis of existing rare moderate-risk alleles associated with cancer (allele frequencies below 1% and ORs 2.0–17.6). Seven of the variants were missense mutations in seven different genes, *RAB11FIP5*, *POTEA*, *COL27A1*, *MUC5B*, *PSMA8*, *MYH7B* and *PABPC1L*, and one was in the downstream region of *NEU1* (Tables 1 and 2).

None of these eight SNPs were found in our previous study [18]. This is likely due to the fact that the first study used unselected cases and the second mostly familial cases, thus more likely to demonstrate rare moderate-risk alleles. The suggested SNPs were mostly exonic and could constitute pathogenic SNPs. However, it is still possible that the SNP is only associated with another pathogenic SNP in its vicinity, and probably targeting the same gene and if so, it cannot be replicated easily using different populations. Findings from a study using mixed populations should be more likely able to be replicated in any population, and, in fact, we in the current study managed to confirm 27 of 30 suggested SNPs from our first paper (Table 3). Another reason for not observing the eight SNPs in the previous study could be that these eight SNPs were not successfully genotyped within the first study. Studies using very rare SNPs are prone to genotyping errors and artefacts, and in fact many more SNPs were suggested but ruled out in our study leaving eight out of 39 from primary analysis.

Both cases and controls in this study were analyzed using Illumina Infinium BeadChip, but on different platforms at different time points. We designed the study by only using SNPs successfully genotyped on both genotyping platforms. In general, genotypes were called by GenCall algorithm in both datasets, however, an additional algorithm zCall was integrated for rare variant calling in the control dataset but not the case dataset. In order to eliminate false risk variants due to incorrect calling, we sequenced all cases as carriers for the suggested risk SNPs. Calling artefacts were observed in most of the suggested SNPs, leaving only eight (25%) suggested moderate risk genetic variants validated. This implicates the challenging nature of rare variant calling.

Accurate estimation of population allele frequency for rare variants depends particularly on representative population and adequate sampling. Although combining data could be used to reduce variance and improve estimation, pooling of samples representing distinct populations can lead to biased estimates. Difference in MAF between European population and all populations in the 1000Genomes [19] and ExAC [20] databases indicates the divergence among populations (Table 1). The SweGen project [31] included 1 000 individuals reflecting a cross-section of the Swedish population, but its sample size could be insufficient for estimation of rare allele frequency. In this study, we used 12 108 unaffected individuals from the Swedish Twin Registry as controls, likely providing a better representation of the Swedish population. This could possibly explain that for some candidate SNPs, the risk allele frequency in CRC cases is not substantially higher than that in SweGen (Table 1).

The variant rs201302413, had the highest OR, 17.6, and was in a gene not well studied. The gene, *PABPC1L*, is known to be involved in transcription and has been shown to be down-regulated by preoperative radiotherapy in rectal cancer [32]. Single cell sequencing in one CRC showed one clone with a *PABPC1* mutation together with a *CDC27* mutation [33].

The variant rs148220987 in the *RAB11FIP5* gene had an OR of 6.3. The Rab GTPases family regulates intracellular membrane trafficking in eukaryotic cells and is known to be involved in cancer signaling pathways [34]. Rab11 regulates JNK and Raf/MAPK-ERK signaling pathways [35] and Rab1 and Rab11 are playing a key role in Notch signaling via vesicular trafficking [36]. Recent discoveries have demonstrated a family of genes downstream of the Rab GTPase, the FIP family [37]. *RAB11FIP5* or *Rip11*, is one of them. FIP function is not well known but shown to be involved in crucial cellular physiological processes such as cell division, and cell migration in various human cancers [37].

The SNP rs754511 in the *MYH7B* gene had an OR of 3.6. This gene is not well studied in CRC but has been studied in melanoma, and *MYH7B* SNPs have been associated with increased melanoma risk [38–40]. It did not segregate well in family 340 (Fig 1).

rs200554635 in the *MUC5B* gene had an OR of 3.5. Stromal genes such as mucins have been shown to affect carcinogenesis, and polymorphisms in microRNA binding sites of those genes have been suggested to predict clinical outcome in CRC patients [41]. Mutations in gene *GALNT12* have been found in colonic cancers and also suggested to predict CRC and this enzyme is initiating mucin type O-linked protein glycosylation and may contribute to a subset of colon cancers [42]. Moreover, Cox2 is well known to be involved in CRC carcinogenesis and has been suggested to act by inducing Muc5B and Muc17 secreting cells in the pathogenesis of esophageal cancer [43].

The SNP rs12193504 is located close to the *NEU1* gene, which has been studied extensively in cancer and suggested to have a profound effect in human cancers [44]. The gene plays a role in sialidase-mediated regulation of tumorigenesis including growth factor receptor signaling, control of TOLL-like receptor signaling and immune-mediated tumorigenesis, regulation of epithelial mesenchymal transition as well as acquired chemo-resistance and regulation of vascularization [44]. NEU1 has specifically been suggested to suppress metastasis in human colon cancer cells [45]. This *NEU1* related SNP is located within transcription factor binding sites and DNase hypersensitivity clusters, suggesting its potential regulatory function. This marker could be tested and found in one additional patient with stomach cancer in family 1164 and in one other CRC patient in family 275, but not in another person with a high-risk adenoma from the same family (Fig 1).

We detected variants of rs202238834 in three of the cases, leading to an allele frequency of 0.001, which is higher than the MAF of 0.0001 from the ExAC project. None of the controls had this variant. This variant is located in the exonic region of *POTEA* gene, member of a highly homologous gene family expressed in a wide variety of human cancers (colon, lung, breast, ovary and pancreas) [46]. Biological function of this gene family is not clear, but there is evidence for its role in inducing programmed cell death [47].

The SNP rs34578955 has a MAF of 0.03%-0.1% in European people, compared to allele frequency 0.14% in our cases. This SNP is within the *COL27A1* gene. Collagens are stromal genes known to be involved in carcinogenesis in experiment animal models [48] and suggested to influence epithelial cells and tumor growth by influences from stromal cells [49]. We have previously suggested that expression of *COL11A1* and *COL5A3* in CRCs could be associated with CRC carcinogenesis [50, 51]. This SNP segregated in three patients with CRC over three generations (family 1213) supporting the SNP to be involved in all family members with CRC in this family (Fig 1). Furthermore, another variant in the splice region was found when the locus was searched for additional markers with suggestive p-value (3E-05) (Table 4).

The risk allele of rs137990346 occurred with a 0.14% frequency among our cases and none in controls, which is reasonable considering the expected frequency in SweGen, ExAC and 1000Genomes. The SNP lies within the *PSMA8* gene, a specific subunit involved in histone acetylation and could play a critical role in chromatin remodeling, DNA repair and epigenetic regulation of gene expression [52].

Moreover, we also searched for additional candidate markers located close to the eight risk variants. Three more SNPs, rs202114540, rs140849642 and rs201749293 were found within 1 Mb from rs148220987, rs34578955 and rs201302413, respectively, and verified by Sanger sequencing (Table 4). Each of these three variants was observed in two unrelated CRC cases, but not in any controls, giving a p-value of 3E-05 which is suggestive for risk variants. SNP rs140849642 is located in the splice region of gene *COL27A1*, confirming the involvement of *COL27A1*, whereas markers rs202114540 and rs201749293 are in the coding regions of *ALMS1* and *HNF4A*. Mutations in *ALMS1* (centrosome and basal body associated protein) gene are known to cause Alström’s syndrome [53, 54]. Several GWAS also reported variants in or near *ALMS1* to be associated with chronic kidney disease [55, 56]. *HNF4A* (hepatocyte nuclear factor 4 alpha) encodes a nuclear transcription factor regulating metabolism, cell junctions, differentiation and proliferation in liver and intestinal epithelial cells. Expression profile of its isoforms has been demonstrated modified in many cancers including CRC [57], and it was implicated that the interaction between Src tyrosine kinase and HNF4α has important implications for colon and other cancers [58].

In conclusion, we suggested eight novel candidate CRC risk loci, within the Swedish population. The study suggest that low frequent risk alleles contribute to the risk of cancer in seemingly high-risk families and also that the total risk in these families are contributed by more than one risk factor such as in complex disease.

## Supporting information

S1 Fig Populational plots for individuals included in the association analysis. Plots from multidimensional scaling (panel A, dimension 1 vs. dimension 2) and principal component analysis (panel B, PC1 vs. PC2) of cases and controls remained for the association analysis.
(PDF)
