# Supplementary figures and images for: Recurrent, low-frequency coding variants contributing to colorectal cancer in the Swedish population
Source: PLoS One. 2018 Mar 16;13(3):e0193547. doi: 10.1371/journal.pone.0193547 (PMC5856271; doi:10.1371/journal.pone.0193547)

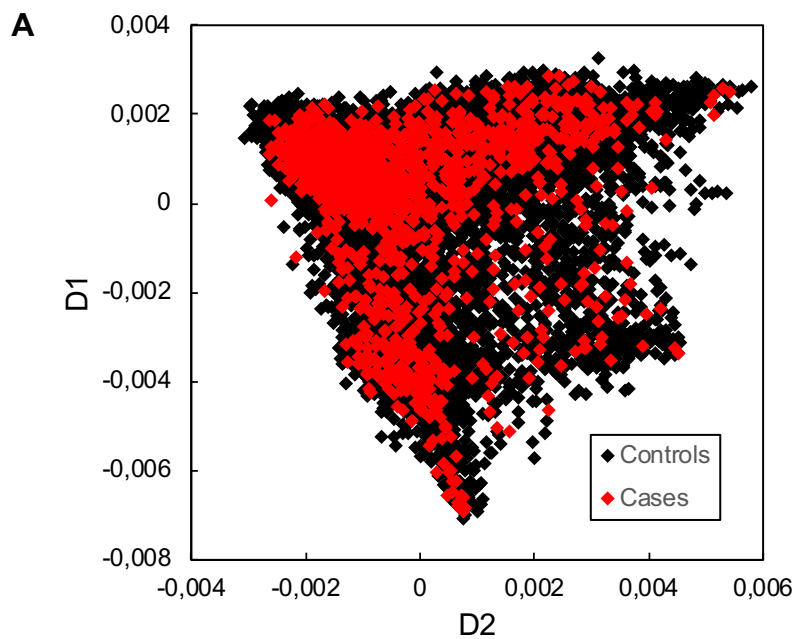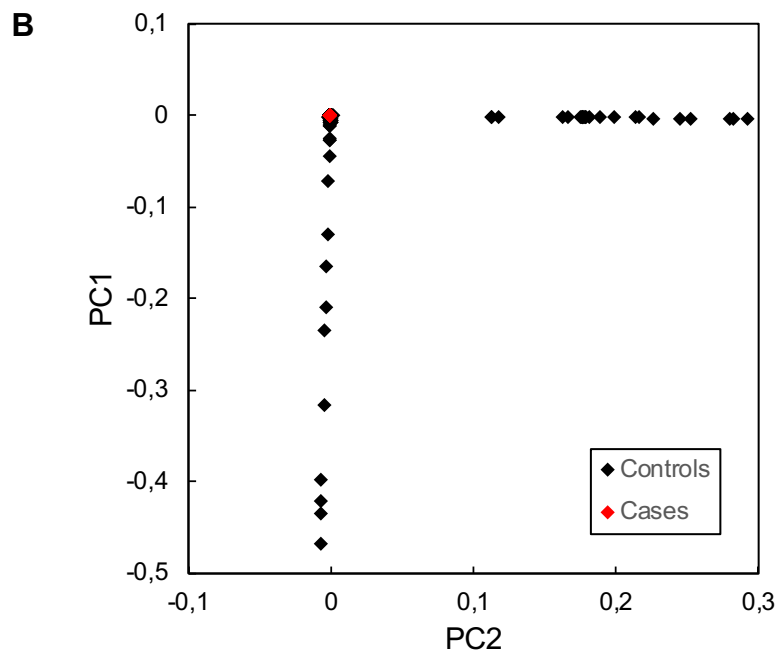

Supplement: S1 Fig — Plots from multidimensional scaling (panel A, dimension 1 vs. dimension 2) and principal component analysis (panel B, PC1 vs. PC2) of cases and controls remained for the association analysis. (PDF) [file pone.0193547.s001.pdf]
